# Affordance Matching Predictively Shapes the Perceptual Representation of Others’ Ongoing Actions

**DOI:** 10.1037/xhp0000745

**Published:** 2020-05-07

**Authors:** Katrina L. McDonough, Marcello Costantini, Matthew Hudson, Eleanor Ward, Patric Bach

**Affiliations:** 1 School of Psychology, University of Plymouth; 2 Department of Psychological, Health, and Territorial Sciences and Institute for Advanced Biomedical Technologies, Gabriele d’Annunzio University of Chieti-Pescara; 3 School of Psychology, University of Plymouth

**Keywords:** action understanding, action prediction, social perception, predictive processing, representational momentum

## Abstract

Predictive processing accounts of social perception argue that action observation is a predictive process, in which inferences about others’ goals are tested against the perceptual input, inducing a subtle perceptual confirmation bias that distorts observed action kinematics toward the inferred goals. Here we test whether such biases are induced even when goals are not explicitly given but have to be derived from the unfolding action kinematics. In 2 experiments, participants briefly saw an actor reach ambiguously toward a large object and a small object, with either a whole-hand power grip or an index-finger and thumb precision grip. During its course, the hand suddenly disappeared, and participants reported its last seen position on a touch-screen. As predicted, judgments were consistently biased toward apparent action targets, such that power grips were perceived closer to large objects and precision grips closer to small objects, even if the reach kinematics were identical. Strikingly, these biases were independent of participants’ explicit goal judgments. They were of equal size when action goals had to be explicitly derived in each trial (Experiment 1) or not (Experiment 2) and, across trials and across participants, explicit judgments and perceptual biases were uncorrelated. This provides evidence, for the first time, that people make online adjustments of observed actions based on the match between hand grip and object goals, distorting their perceptual representation toward implied goals. These distortions may not reflect high-level goal assumptions, but emerge from relatively low-level processing of kinematic features within the perceptual system.

## Public Significance Statement

This study shows that our perceptions of others’ actions are biased by which object we expect the person to reach for. Hands that form a large grip are seen to reach closer to large objects than they really were, and hands that form a small grip are seen to reach closer to small objects. These action expectations are generated during the ongoing observation of other’s actions, as grip information becomes available, and is matched to the size of objects within reaching distance. These expectations distort the perception of the action, and are independent from explicit action evaluations, suggesting that they emerge from processes within the perceptual system itself, rather than from more sophisticated, high-level reasoning about the actor’s goals.

The ability to understand and predict other people’s behavior is a cornerstone of human social cognition and makes people’s sophisticated interactions with others possible. Parents rapidly intervene when their child reaches for a hot cup of coffee instead of a toy. In sports, players anticipate each other’s behavior, fluently passing a ball to a teammate’s future position. In contrast, deficits in the ability to understand others’ behavior are a hallmark of several conditions that bring with them marked impairments in social interactions, such as autism (e.g., Koster-Hale & Saxe, 2013; Pellicano & Burr, 2012; von der Lühe et al., 2016).

These abilities for social perception are conventionally conceptualized as a simple bottom-up process, in which incoming visual information about others’ behavior is matched to one’s higher-level motor—or conceptual—knowledge about it, so that the action’s meaning and associated mental states can be derived (Gallese & Goldman, 1998; Rizzolatti & Sinigaglia, 2010; Rizzolatti, Cattaneo, Fabbri-Destro, & Rozzi, 2014). However, there is no one-to-one mapping between stimuli and meaning that such a mechanism could rely on (Ansuini, Cavallo, Bertone, & Becchio, 2015; Jacob & Jeannerod, 2005; Uithol, van Rooij, Bekkering, & Haselager, 2011). The same behavior can mean multiple things in different contexts (e.g., a smile), and the same goals can be accomplished by multiple behaviors (e.g., closing a drawer with one’s hand vs. hip).

It has therefore been argued that social perception is better understood as a predictive process in which the brain constantly tests hypotheses about the observed action against the perceptual input (e.g., Bach, Nicholson, & Hudson, 2014; Bach & Schenke, 2017; Csibra, 2008; Donnarumma, Costantini, Ambrosini, Friston, & Pezzulo, 2017; Kilner, Friston, & Frith, 2007a, 2007b). In such accounts, any assumption about others’ goals and beliefs—derived perhaps from prior knowledge about the individual (e.g., Joyce, Schenke, Bayliss, & Bach, 2016; Schenke, Wyer, & Bach, 2016) or from contextual information (e.g., objects, Bach, Knoblich, Gunter, Friederici, & Prinz, 2005; Jacquet, Chambon, Borghi, & Tessari, 2012; Kalénine, Shapiro, Flumini, Borghi, & Buxbaum, 2014; Nicholson, Roser, & Bach, 2017; gaze and emotional expressions, Adams, Ambady, Macrae, & Kleck, 2006; Frischen & Tipper, 2006)—is translated into predictions about which behavior should be observed if these assumptions were correct and is superimposed over the perceptual input. Such an integration would not only help to stabilize perception, filling in gaps in the input (e.g., in the case of occlusion, Prinz & Rapinett, 2008) or compensate for the considerable noise during motion perception (Hammett, 1997), but would also let mismatching behavior of others’ stand out, so that our assumptions about them can be revised until they better explain their behavior.

We recently developed an experimental paradigm that can make these predictions visible (Hudson, Bach, & Nicholson, 2018; Hudson, McDonough, Edwards, & Bach, 2018; Hudson, Nicholson, Ellis, & Bach, 2016; Hudson, Nicholson, Simpson, Ellis, & Bach, 2016; McDonough, Hudson, & Bach, 2019). This paradigm rests on the assumption that, if predictions indeed act on perceptual representations (Bar, 2004; de Lange, Heilbron, & Kok, 2018; Ekman, Kok, & de Lange, 2017) then every prediction one makes about another person may subtly bias the perception of this person’s forthcoming actions, especially in the case of uncertainty, such as the visual blurring during motion perception. Thus, in the same way as prior expectations in the nonsocial world cause us to see a color differently (Bloj, Kersten, & Hurlbert, 1999; see for an application to the blue/gold dress illusions, Chetverikov & Ivanchei, 2016) or shapes as either convex or concave based on the surrounding illumination (Adams, Graf, & Ernst, 2004), our prior knowledge of other people—their goals and intentions—may subtly shape the perceptual experience of their actions, and help us plan our own response toward it (see Brouwer & Knill, 2007, 2009 for an example in nonsocial perception).

This is indeed what we observed in a series of studies. Participants heard an actor make a statement about his goal— “I’ll take it” or “I’ll leave it”—before they briefly saw him start to either reach for an object or withdraw from it, irrespective of what the actor said. The action disappeared midmotion and participants indicated the perceived vanishing point, either by comparing it to a probe stimulus shortly after stimulus offset (Hudson, Nicholson, Ellis et al., 2016; Hudson, Nicholson, Simpson et al., 2016) or by indicating its disappearance point on a touch screen (Hudson, Bach et al., 2018; Hudson, McDonough et al., 2018). The results consistently revealed predictive biases on these perceptual judgments. First, hands were generally reported to have disappeared further along the trajectory than what was actually seen (i.e., closer toward the object for reaches, further away for withdrawals), capturing predictions derived from extrapolating the action’s prior kinematics (i.e., the classical representational momentum effect, Freyd & Finke, 1984; Hubbard, 2005). Second, and more importantly, they revealed an influence of goals attributed to the actor: hands were reported to have disappeared further toward the object when the actor said they would take it and further away from the object when the actor said they wanted to leave it.

Since then, other studies extended these findings, showing that similar distortions can be induced when the participant instructed the observed actor (e.g., telling the actor to “take it!” or “leave it!” before action onset, Hudson, Nicholson, Ellis et al., 2016) and that the observed actor’s long-term reliability to do as they said modulates the strength of the prediction effects (Hudson, Bach et al., 2018). Most recently, we showed that similar effects can be elicited by the prior object context, such that hands reaching straight for an object are perceptually judged to veer slightly upward if they would need to reach over an obstacle, and slightly downward when reaching unnecessarily high (Hudson, McDonough et al., 2018). Strikingly, the same biases are not observed for inanimate objects (e.g., a ball) that move on closely matched trajectories (McDonough et al., 2019), linking the perceptual biases to attributions of agency and intentionality to the observed stimulus.

These data show that the goals attributed to others are indeed translated into predictions about their upcoming action, which then bias perceptual judgments toward these expectations. Yet, in all these studies the goals or environmental constraints were explicitly given prior to action onset. In the real world, people typically do not always announce their intentions before their action. Instead, an action’s goal often has to be derived dynamically once the action is underway and its kinematics become apparent (Ambrosini, Costantini, & Sinigaglia, 2011; Ambrosini et al., 2013; Bach, Bayliss, & Tipper, 2011; Sartori, Becchio, & Castiello, 2011).

We have argued that the affordances of the goal objects could play a major role in this process (Bach et al., 2014). For example, it is well-known that during natural reaching, the hand forms (“preshapes”) a grip that matches the intended target object, long before the object is reached. Viewing an action (e.g., a reach with small grip aperture) that matches—in terms of trajectory, kinematics, and hand grip, for example—the affordances of an available target object (e.g., a small object like a strawberry or grape) would immediately signal to an observer what the goal of the action would be (e.g., Bach et al., 2005; Bach et al., 2011; van Elk, van Schie, & Bekkering, 2014). And indeed, there is now ample evidence that people spontaneously derive the target of a reach, by matching the hand’s grip configuration—that is, either a small “precision” grip or a large “power” grip—to the available large or small objects in the environment (e.g., Ambrosini et al., 2011, 2013; for a review, see Bach et al., 2014). For example, eye movements reveal that people anticipate the target of an ongoing reach by matching the unfolding grip shape (large or small grip) to the surrounding objects before the action is completed (e.g., Ambrosini et al., 2011, 2013), automatic imitation effects are larger for actions that match a goal object (e.g., Bach et al., 2011) and larger motor evoked potential are elicited by the same kinematics if they match an available goal object (Southgate, Johnson, El Karoui, & Csibra, 2010).

Here we test, for the first time, whether people use such grip–object matching not only to derive the action’s goal or target (e.g., which goal object is selected), but whether they also use these goals, as assumed by perceptual prediction models (e.g., Kilner et al., 2007a, 2007b), to predict how an action is assumed to develop, even if it is already well underway. If this is the case, we should find that the match of an unfolding hand grip to one of two objects in the environment should again induce such perceptual biases, and they should be measurable—as in our prior work—in subtle distortions in perceptual judgment about these actions. Demonstrating such distortions is crucial to show that, during action observation, people go beyond simple goal inference (e.g., identifying the target of a reach) but that they use this information to predict which future course the action will take to achieve these goals, which has only rarely been tested in prior research.

In two experiments, we presented participants with brief videos of an actor’s hand starting at rest on the right side of a table, with two potential goal objects—one large, one small—next to each other on the left, one closer in the foreground and the other further into the background. The hand then begins to reach toward the two potential target objects, with the hand forming either a whole-hand power grip or a precision grip, therefore matching one of the two objects but not the other. The hand disappeared midmotion, at a roughly equal distance—in 3D space—away from either object, and participants were required to indicate the final location of the hand’s index finger on a touchscreen monitor. If observers identify the goals of the action by matching the observed grip to the two objects’ affordances and then form a perceptual prediction about its future course, then perceptual judgments should show specific biases: the located disappearance points should be reported closer to the corresponding object than they actually were, and away from the alternative (mismatching) target object. Therefore, although the hand actually reached with the same trajectory between the two objects, reaches with a precision grip should be reported closer to the smaller object and reaches with a power grip should be perceptually biased toward the larger object.

A crucial question is whether any such effects emerge from a general top-down mechanism, such that high-level attributions of others’ goals penetrate lower-level perceptual representations, or whether any perceptual biases emerge from “encapsulated” interactions in the perceptual system itself (Firestone & Scholl, 2016; Scholl & Gao, 2013), which has already been shown to be sensitive to such matching hand–object interactions (e.g., Bracci & Peelen, 2013). We assumed that the perceptual biases in our prior research (Hudson, Nicholson, Ellis et al., 2016; Hudson, Nicholson, Simpson et al., 2016; Hudson, Bach et al., 2018) emerged from high-level information, when people either heard the person make goal statements (“I’ll take that!”) or instructed them about the appropriate action (“Take it!”). However, in these studies, these goals were given well in advance of the action commencing, so that participants had ample time to “tune” lower-level processes toward the expected goal. To test whether such high-level information can penetrate online social perception—when the action’s goal only becomes apparent while the action is underway—we manipulated, across the two experiments, whether participants had to explicitly derive the action’s goals or were given no such instruction. In Experiment 1, we had participants say into the microphone, after each trial, which object they thought the hand was reaching for, therefore requiring explicit goal monitoring in each trial. In Experiment 2, no such verbal responses had to be given but participants still reported the perceived disappearance points. These localization judgments therefore measure spontaneous goal inferences and the resulting predictions.

These two experiments allow us (a) to replicate any observed biases toward the implied goal object in two separate samples, and (b) to tease apart from which kind of mechanisms these biases emerge. If perceptual biases emerge from a more or less encapsulated, automatic visual prediction system that relies on perceptually available “local” stimulus features (e.g., precision vs. power grip, available objects; for a review, see Scholl & Gao, 2013) then they should be observed—and of roughly equal size—both when the instruction asks participants to view the actions in a teleological manner (Experiment 1) or not (Experiment 2). In contrast, if these biases require attentional focus on the action’s goals, then they should be larger—or only observed—in Experiment 1 where such a focus was induced. Moreover, the combination of explicit verbal goal judgments and implicit perceptual judgments in Experiment 1 will allow us to test, across participants and across trials, the relationship between these measures.

## Method

### Participants

Sixty-two participants took part in Experiment 1 (mean age = 20 years, *SD* = 3.4, 52 females) and 63 participants took part in Experiment 2 (mean age = 21 years, *SD* = 5.5, 50 females). Eleven additional participants across both experiments were excluded due to performance assessed against several a priori criteria (see Results) established in our prior research (Hudson, McDonough et al., 2018; McDonough et al., 2019). All were right-handed and had normal/corrected-to-normal vision, and were recruited from University of Plymouth for course credit. The study was approved by the University of Plymouth Ethics Committee, in accordance with the declaration of Helsinki.

Power analyses were conducted with G*Power (Version 3.1) using the sensitivity analysis function. We do not report theoretical power based on previously reported effect sizes as this neglects uncertainty around these effect size measurements (e.g., Anderson, Kelley, & Maxwell, 2017). Instead, we report effect sizes that can in principle be detected with our experimental parameters (i.e., given required power, participant numbers and type of test). This analysis revealed that a sample size of 62 provides .80 power to detect effects in the predicted direction with Cohen’s *d* = .32, and effects in either direction with Cohen’s *d* = .36. Our prior studies investigating similar effects with the same method (Hudson, Bach et al., 2018; Hudson, McDonough et al., 2018; McDonough et al., 2019) revealed that effect sizes are consistently larger (*d* = .52 to *d* = 1.23). Effect sizes are expected to be smaller here as, contrary to these prior studies, predictions could only be formed when the action was underway and the prediction-relevant stimulus features were not mentioned to participants before the experiment started. Indeed, a small-scale pilot study with the same design as Experiment 1 showed that the effects would be present, but with a smaller effect size (*d* = .39).

### Apparatus

Presentation (NeuroBS) software was used to present the experiment via a HP EliteDisplay S230tm 23-in. widescreen (1920 × 1080) Touch Monitor. Verbal responses for Experiment 1 were detected using Presentation’s Sphinx speech recognition engine via a Microsoft LifeChat LX-3000 Headset.

### Stimuli

**Figure 1 fig1:**
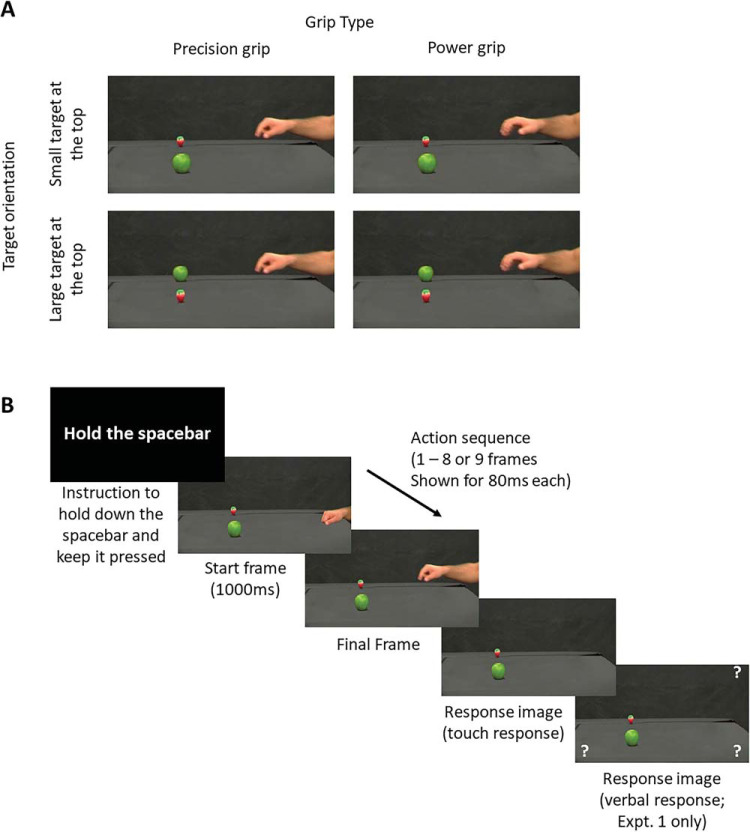
Panel A: Experimental conditions and trial sequence. The objects were arranged with either the small object (strawberry) on top and the large object (apple) on the bottom (top row), or with the large object on top and the small object on the bottom (bottom row). The actor’s hand reached with either a precision (small) grip (left column), or with a power (large) grip (right column). Panel B: Example of a trial sequence, showing a “small object on top” configuration with a small grip.

Example stimuli can be seen in Figure 1A. Stimuli were derived, using photo manipulation, from a prior stimulus set of video stimuli from one of the authors (Costantini, Ambrosini, & Sinigaglia, 2012). The videos (950 × 540) showed an actor’s arm, from a slightly elevated side view, reaching over a black table from the right toward two potential target objects adjacent to each other in depth on the left side of the table: a small target (a strawberry) and a large target (an apple). They were derived from videos of natural (e.g., nonpantomimed) reaches toward one of the two objects, which were then photo-edited such that both objects were located at an equal distance (in 3D space) away from the hand, with one closer to the foreground and therefore appearing lower down on the screen and one closer to the background and higher up on the screen (object positions counterbalanced across trials). The two objects were chosen because both their size and typical use clearly affords small “precision” grips (strawberry) and large whole-hand “power” grips (the apple), but they have similar abstract round shapes and merely differ in color, so that they represent prototypical objects. Both objects cast a small shadow on the surface underneath, reinforcing the impression that they were resting on the table and were separated in depth.[Fig-anchor fig1]

The actor’s hand started at rest in a neutral closed hand posture, and then began to reach, with the hand progressively opening to form either a whole-hand power grip or a thumb-index precision grip. Four reach videos were used for each hand preshape condition (i.e., precision vs. power grip), which together with two target layouts (small object to the front, large at the back, or vice versa), created a total of 16 different videos. Each video was converted into nine frames, where Frames 8 and 9 showed the actor’s hand at the approximate point of maximal grip aperture for both the power and precision grip sequences, roughly halfway between the starting position and the objects, and at a position in depth between the two objects, so that only whether a precision or power grip was formed would predict which object would be reached for and not the hand position. The shadow of the hand was digitally removed so this information could not aid localization in depth.

Response images for both experiments were created by digitally removing the actor’s arm and hand from the scene, so that only the target objects and the background remained. Presenting this frame immediately after the action sequence gave the impression of the hand disappearing from the scene. A second response image for Experiment 1 was identical to these images, with the addition of four question marks positioned at each corner of the screen. These served as cues for participants to make their verbal responses about which object they believed was the target. All editing was completed using Adobe CC Photoshop.

### Procedure

An example trial sequence can be seen in Figure 1B. Participants completed a total of 192 trials, consisting of four blocks of 48 trials (each representing all 16 different trials three times), with breaks in between. At the start of each trial, participants saw an instruction to “Hold the spacebar,” to which they pressed the spacebar with their right hand and kept it depressed until the end of the action sequence. They were instructed to keep their left hand in their lap at all times, and this was constantly monitored throughout the experiment. This ensured that they did not track the observed action with their finger, and could only initiate their response with the right hand once the action sequence had disappeared. They then saw the first (neutral) frame of the action sequence for 1,000 ms, followed by successive frames at 80-ms intervals. To increase variability of the hand’s final position, the final frame was randomly chosen as either Frame 8 or Frame 9, in which the hand’s intended grip posture was fully visible. This final frame was then immediately replaced with the response image. Participants released the spacebar and, with their right hand, touched the screen where they thought the final position of the tip of the observed index finger was (see Hudson, McDonough et al., 2018; Ashida, 2004; Motes, Hubbard, Courtney, & Rypma, 2008, for similar procedures). In Experiment 2, the next trial began as soon as the touch response was registered. In Experiment 1, the touch response was immediately followed by the second response frame where participants were required to say into the microphone which target object they thought the actor was reaching toward (either apple or strawberry). Once the verbal response was registered, the next trial began. The placement of the explicit responses at the end of the action sequences, after perceptual judgments, ensures that explicit verbal responses do not interrupt perceptual judgments, and that the methodology across Experiments 1 and 2 match as closely as possible and can be directly compared.

## Results

Data were collected in two separate experiments, but for brevity and due to their methodological similarity, we analyze them together in one omnibus ANOVA, with experiment entered as a between-subjects variable. Further separate analyses are presented for each experiment to demonstrate the robustness of the effects.

### Data Preprocessing

Data filtering was identical to Hudson, McDonough et al. (2018) and McDonough et al. (2019). In both experiments, individual trials were excluded if the correct response procedure was not followed (e.g., lifting the spacebar before the response image was presented; 2.8% of total trials), or if response initiation or execution times were less than 200 ms or more than 3 *SD* above the sample mean (2.4%, Initiation: *M* = 355.5ms, *SD* = 143.7; Execution: *M* = 646.0 ms, *SD* = 240.0). Participants were excluded if too few trials remained after trial exclusions (<50% trials, 5 participants), if their average distance between the real and selected positions was more than 3 *SD* away from the sample mean (*M* = 39.0 pixels, *SD* = 17.0, 2 participants excluded), or if the correlation between the real and selected positions was more than 3 *SD* below the median r value (X axis: median *r* = .762, *SD* = .113; Y axis: median *r* = .860, *SD* = .098, 2 participants excluded). Two further participants were excluded from Experiment 1, one because they selected the top object as the most likely target object in *all* trials, compared to the remaining participants (top object selected in 51% of trials), and one for showing an abnormally large perceptual bias in the predicted direction (i.e., 15 times larger than the sample mean) so that we suspected a misunderstanding of the task (e.g., touching the likely target object instead of the hand disappearance point). Removal of these two participants does not affect the results. This left a total of 62 participants in Experiment 1 and 63 participants in Experiment 2.

### Perceptual Biases

**Figure 2 fig2:**
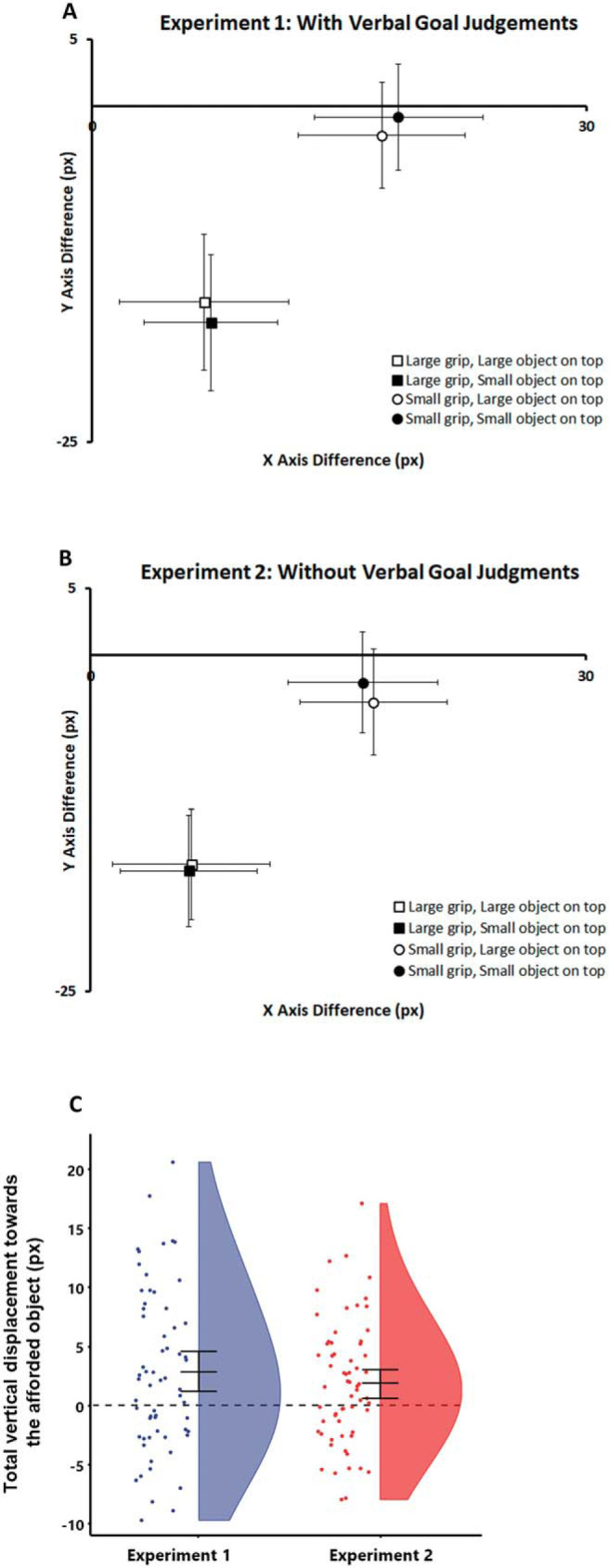
Panel A: Grip Type × Object interaction for Experiment 1. The difference scores between the real final position and the selected final position is plotted for the X axis and Y axis. Panel B: Grip Type × Object interaction for Experiment 2. Panel C: A raincloud plot (Allen, Poggiali, Whitaker, Marshall, & Kievit, 2018) of the comparison across experiments of the size of the Y axis interaction in pixels, equivalent to the total amount by which each grip type was distorted toward the congruent object (mathematically equivalent to the O displacement in representational momentum studies, Hubbard, 2005; Hubbard & Bharucha, 1988). Each data point represents this Y axis interaction value for each participant. Error bars in all plots depict 95% confidence intervals.

Analysis was conducted on the perceptual bias, which reflects the difference between the hand’s real disappearance point and participants’ subjective judgments on the X and Y axis in screen coordinates. As in our prior research that established the procedure (Hudson, McDonough et al., 2018; McDonough et al., 2019), it was derived by subtracting the real final coordinates of the tip of the index finger from the participant’s selected coordinates on each trial (see Figure 2). This resulted in separate difference scores for the X and Y axis, where positive X and Y scores represented a rightward and upward displacement respectively, and negative X and Y scores represented a leftward and downward displacement respectively. A score of 0 on both axes indicated that the participant selected the real final position exactly. The coding of negative and positive values on both axes matches the usual convention of screen coordinates. It therefore allows us to represent deviations in localizations as they would appear on the touch screen, relative to the actual disappearance points, without requiring further transformations^1^ (see Figure 2A and 2B).[Fig-anchor fig2]

#### Y axis

Participants’ perceptual biases on the Y axis were analyzed with a 2 × 2 × 2 mixed ANOVA, with Grip type (power vs. precision) and Object location (large target on top vs. small target on top) as repeated measures factors and Experiment (1: explicit prediction vs. 2: implicit prediction) as between-subjects factor. We predicted, first, that the perceived disappearance points of reaches would be distorted toward their apparent target object, anticipating the action’s predicted future course. As a consequence, reaches should appear to have terminated slightly higher if they matched a target object at the top and lower for a target object at the bottom. Second, if these perceptual biases depend on viewing the actions teleologically, in terms of their goals, then these shifts should be larger, or only observed, in Experiment 1, where the hands’ goals were task-relevant, compared to Experiment 2 where such goal inferences would only occur spontaneously. These effects should be reflected in a two-way interaction of grip type and object location and a three-way interaction of grip type, object location, and experiment. Note that each participant’s contrast value for the two-way interaction of grip type and object location is mathematically identical to their overall bias in judgments toward the target object on the Y-axis, as typically reported in studies on representational momentum (O displacement, Hubbard, 2005; Hubbard & Bharucha, 1988). We present means and single data points on this summary measure for each participant in raincloud plots (Allen, Poggiali, Whitaker, Marshall, & Kievit, 2018) for both experiments in Figure 2C.

As can be seen in Figure 2A and 2B, disappearance judgments were generally lower than the real position, consistent with previously reported shifts of localization responses toward the object’s center of gravity in touch screen studies (e.g., Coren & Hoenig, 1972; Hudson, Bach et al., 2018; McDonough et al., 2019) and a general downward bias in localization responses in representational momentum-like paradigms (i.e., representational gravity, Hubbard, 2005).

The omnibus ANOVA revealed the predicted interaction of grip type and object location, *F*(1, 123) = 16.8, *p* < .001, η_p_^2^ = .120, BF10 = 158. Even though kinematics were identical, the disappearance point of power grips was reported higher when the large target object was placed at the top (−15.1 px) than when the large target object was placed at the bottom (−16.0 px, *t*(124) = 2.75, *p* = .007, *d* = .25). Conversely, the disappearance point for precision grips was reported to be higher when the small target object was at the top (−1.5 px) compared to when the small target object was at the bottom (−2.8 px, *t*(124) = 3.64, *p* < .001, *d* = .33). Note that while these deviations are small, they are highly reliable, reflected in a Bayes factor of BF10 of 158 for the interaction, and—especially when their smaller than lifelike representation on the monitor is considered—surpass similar deviations induced by object biases in the kinematics of real reaches (e.g., distractor interference/deviation ≈ 1 mm, Keulen, Adam, Fischer, Kuipers, & Jolles, 2003; Welsh, Elliott, & Weeks, 1999).

The second question was whether the size of these perceptual displacements was larger when the actions’ target was task relevant in Experiment 1 compared to when it was task-irrelevant in Experiment 2. However, there was no three-way interaction between grip type, object location and experiment, *F*(1, 123) = .666, *p* = .416, η_p_^2^ = .005, BF10 = .263. Moreover, the relevant interaction of grip and object location was present in both experiments, irrespective of whether participants explicitly reported the action’s goals after the perceptual judgments (Experiment 1: *F*(1, 61) = 9.56, *p* = .003, η_*p*_^2^ = .135, BF10 = 30.2, Experiment 2: *F*(1, 62) = 7.20, *p* = .009, η_*p*_^2^ = .104, BF10 = 11.0, see Figure 2). Two one-sided tests (TOST) procedure (Lakens, 2017) indicated that the observed effect size of the between-experiment difference (*d* = .16) was significantly within the equivalence bounds of ΔL = –.51 and ΔU = .51, *t*(113.89) = −1.95, *p* = .027, and a Bayesian analysis provides substantial evidence for the null hypothesis, BF10 = .110.

As all other effects in the ANOVA were not predicted, they should be treated as incidental findings, unless they pass a threshold of *p* < .01, corrected for multiple comparisons in an ANOVA (Cramer et al., 2016). Only the main effect of grip type, *F*(1, 123) = 1130, *p* < .001, η_*p*_^2^ = .902, BF10 = 3.20e + 141, surpassed this threshold, showing perceived disappearance points of power grips were displaced further downward than precision grips. This was expected since the power grip is larger and therefore has a lower center of gravity, which is known to affect touch screen judgments, but is independent from our effects of interest (for similar findings, see Coren & Hoenig, 1972; see also Hudson, Bach et al., 2018; Hudson, McDonough et al., 2018). There were no further main effects or interactions (all *F* < 1.62, all *p* > .205, all BF10 < .472, see the table in the online supplemental material for a report of all effects).

#### X axis

Perceptual biases on the X axis were analyzed with the same ANOVA model. As the two target objects were equidistant from the hand disappearance point on the X axis, the apparent matching of grip to the top or bottom object should not affect deviations on the X axis. All effects in this ANOVA are therefore unpredicted and incidental, and subject to alpha inflation due to multiple comparisons in an ANOVA (Cramer et al., 2016). They should therefore be evaluated against a Bonferroni-adjusted alpha of *p* < .007. Only the main effect of grip type, *F*(1, 123) = 503, *p* < .001, η_*p*_^2^ = .804, BF10 = 1.17e + 99, passed this adjusted threshold, with the perceived disappearance point of power grips more leftward than precision grips, which again reflects the well-known biases in touch screen responses to the more leftward center of gravity for the more spatially isolated index finger in power grips than precision grips. There were no further main effects or interactions (all *F* < 4.07, all *p* > .046, all BF10 < .226, see online supplemental material for details).

### Relationships Between Perceptual Shifts and Explicit Goal Judgments

An important question is to what extent the perceptual displacements measured above are informed by people’s higher-level, explicit judgments about the observed actions. If perceptual predictions are shaped by high-level goal attributions or vice versa, then perceptual displacement and explicit judgments in Experiment 1 should be closely linked, both across participants and across trials within participants.

#### Do people rely on affordance matching to make explicit goal judgments?

We first established whether grip/object matching does not only inform perceptual biases in action observation, but also people’s explicit judgments about the action’s goals. Participants were never given explicit instruction about the relevance of grip/object match, but they reported, after each action in Experiment 1, whether they subjectively experienced the hand to be reaching for the apple or the strawberry. To test whether these judgments were informed by the apparent grip/object match, we separately coded which object the grip actually corresponded to and participants’ subjective judgments about which object they felt the hand reached for. To this end, a hand grip matching the bottom object was coded as 0 and a match to the top object was coded as 1. Verbal goal judgments were similarly coded as 0 and 1 for perceived goal objects at the bottom and at the top, respectively. We then simply, for each participant, calculated the proportion of verbal goal judgments that corresponded to the actual match with the goal object. A simple *t* test against chance (50%) revealed that explicit judgments followed the actual hand–object match (*M* = 66.7%, *SD* = 16.7%; *t* = 31.5, *p* < .001). This confirms that the grip–object match did not only inform perceptual displacements (see main analysis), but also participants’ explicit goal object judgments. It is important to note that even in the explicit judgments grip–object matching only induced a subtle shift toward the intended goal object (i.e., 17% away from chance performance). This is consistent with the fact that grip–object matching is only one of many potential stimulus features (e.g., direction, kinematics) in natural reaches that provides information about the action’s target, which was not specifically mentioned to participants. The shift in explicit judgments therefore measures participants’ spontaneous use of this feature, allowing us to test in how far this explicit use is linked to the measured biases in perceptual judgments, both across participants and across the trials within the experiment.

#### Are perceptual biases and explicit judgments related across participants?

We then tested whether individual differences in participants’ tendency to rely on grip/object matching to make explicit judgments is related to their reliance on grip/object matching in perceptual judgments. We therefore correlated the proportion to which each participant’s verbal goal judgments matched the actual hand/object match with their perceptual biases toward the grip-matching goal object (i.e., the interaction contrast that marks the predictive perceptual shift due to matching grips to object affordances in the main analysis above, mathematically identical to the O displacement in representational momentum studies, Hubbard, 2005; Hubbard & Bharucha, 1988). Surprisingly, the two types of judgments were almost perfectly uncorrelated, *r*(59) = .08, *p* = .518, *N* = 62. TOST procedure (Lakens, 2017) indicated that the observed effect size (*r* = .08) was significantly within the equivalence bounds of ΔL = –.36 and ΔU = .36, *p* = .011. A Bayesian analysis provided substantial evidence for the absence of a correlation, BF10 = .102.

#### Are perceptual biases and explicit judgments related across trials?

While there may be no overall relationships between a participants’ perceptual biases and explicit judgment, it is possible that such relationships are present on a trial-by-trial basis. If explicit and perceptual judgments depend on one another, then actions/trials judged explicitly to be directed toward the top object should also be more likely to show a perceptual mislocation toward the top than the bottom, and vice versa for reaches judged to be directed to the bottom object. As these trial-by-trial relationships are independent of the overall (mean) value on both measures, they can be observed irrespective of any across-participants relationship between perceptual and explicit judgments.

**Figure 3 fig3:**
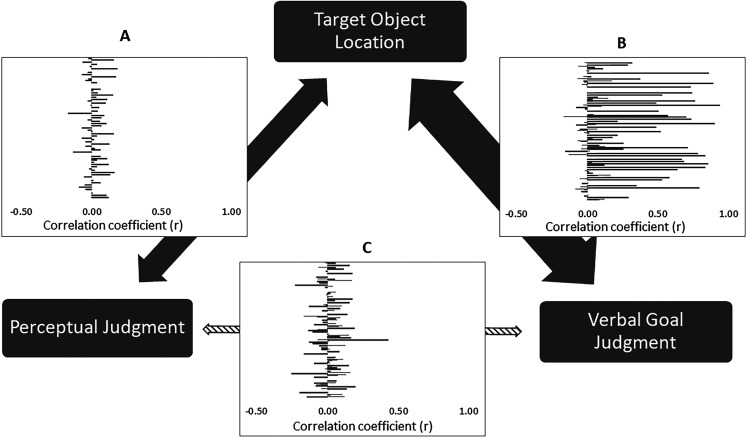
Individual participants’ across-trial correlation coefficient (*r*) between the three measures in interest. The horizontal bars in each of the three panels show each participant’s pairwise across-trial correlation coefficients for the correlation between perceptual judgments and the target object location (top object or bottom object, based on the grip-object match, Panel A), the correlation between verbal goal judgments and the target object location (Panel B), and the correlation between perceptual judgments and verbal goal judgments (Panel C).

To test this, we first verified that the perceptual shifts in each trial reflected the actual target object location. We therefore correlated, for each participant separately, the actual target object location (coded as 0 or 1) for each trial with the size of the perceptual judgment displacement on the Y axis across all trials of the participant. Testing the resulting fisher-transformed correlation coefficients against zero with a simple *t* test revealed a positive mean correlation between perceptual shifts and target object location across participants (mean *r* = .03, *t* = 3.10, *p* = .003; *d* = .40), see Figure 3A. This trial-by-trial correlation was replicated in Experiment 2 (mean *r* = .02, *t* = 2.77; *p* = .007; *d* = .35), and did not differ from Experiment 1, *t*(123) = .512, *p* = .609, *d* = .14. TOST procedure (Lakens, 2017) indicated that the observed effect size between the experiments (*d* = .14) was significantly within the equivalence bounds of ΔL = –.51 and ΔU = .51, *t*(121.5) = −2.06, *p* = .021. As before, a Bayesian analysis of this effect provided substantial evidence for an absence of such a difference, BF10 = .134. Replicating the results of the main analyses with an across-trials correlational measure, this analysis therefore confirms that actions in which the hand grip matched the top object induced larger shifts upward than a grip match to the bottom object.[Fig-anchor fig3]

Next, we performed an identical across-trials correlation analysis for the relationship between the actual target object location and verbal goal judgment. Replicating the finding from the main analysis that explicit judgments follow grip matching, this again revealed a positive correlation between verbal goal judgments and grip information (mean *r* = .44, *t* = 7.20, *p* < .001; *d* = .91), see Figure 3B. Across trials, reaches whose grip matched the top object were therefore more likely to be judged to be reaching to the top object, and vice versa for reaches whose grip matches the bottom object.

Finally, the crucial question was whether explicit verbal goal judgments about an action and the perceptual shifts showed a positive relationship. Strikingly, as in the across-participants analysis, there was no correlation between perceptual displacements and the explicit verbal goal judgments across trials (mean *r* = .01, *t* = .694, *p* = .500, *d* = .09), see Figure 3C. TOST procedure (Lakens, 2017) indicated that the observed effect size (*d* = .09) was significantly within the equivalence bounds of ΔL = –.36 and ΔU = .36, *t*(61) = −2.15, *p* = .018, and a Bayesian analysis provided strong evidence for the absence of a relationship, BF10 = .088. Thus, while the actual target location directly related to the perceptual judgments and to the verbal goal judgments, the two types of judgments were not related to each other. Figure 3 shows participants’ individual across-trial correlation coefficients for the three correlations of interest.

## Discussion

Prior work has shown that people integrate object and action kinematic information to derive the likely goal of observed actions, even while the action is still ongoing (Ambrosini et al., 2011; Bach et al., 2011; Decroix & Kalénine, 2018; Eshuis, Coventry, & Vulchanova, 2009; for a review, see Bach et al., 2014). Here, we tested the hypothesis of predictive processing models (Bach & Schenke, 2017; Hudson, Nicholson, Ellis et al., 2016; Hudson, Nicholson, Simpson et al., 2016) that such goal inferences are immediately translated into perceptual predictions about the actions future path toward the inferred goal, and bias perceptual judgments toward these expected trajectories.

The data from two experiments supported this proposal. In each trial, participants observed the initial stages of a reach toward two potential target objects that differed in size, with either a whole-hand power grip or a precision grip, and were asked to localize the hand’s last seen position after its sudden offset. The results revealed consistent biases in perceptual judgments toward action expectations derived from the match between the emerging grip type and object size. While reaches with a power grip were reported to be closer to large objects, reaches with a precision grip were perceived to be closer to small objects, even when actions with the same kinematics were observed and only the location of the relevant target object changed. These perceptual mislocations were present both when participants were explicitly asked to identify the goal objects in a secondary task (Experiment 1) and when the reach targets were completely task irrelevant and participants were only asked to accurately report the hand’s disappearance point (Experiment 2). Moreover, they were observed even though the actor’s hand started at rest. Which of the two objects was the target object was therefore ambiguous before action onset and only became apparent once the action commenced and a specific grip type began to form.

These perceptual displacements toward the expected kinematics support predictive processing accounts of social perception, which argue that any inferences about an observed action’s goal will (a) give rise to predictions about the action’s further kinematics, which can then (b) bias action perception toward these expectations (Bach & Schenke, 2017; Hudson, McDonough et al., 2018; Hudson, Nicholson, Ellis et al., 2016; Hudson, Nicholson, Simpson et al., 2016; Kilner et al., 2007a, 2007b). They go beyond previous findings in which action expectations were explicitly induced prior to action onset; for example, by asking participants to instruct the (virtual) actor (Hudson, Nicholson, Ellis et al., 2016), by hearing goal statements of the actor (“I’ll take it!”, Hudson, Bach et al., 2018; Hudson, Nicholson, Simpson et al., 2016), or by presenting a static image of the goal object with or without obstructing objects in the way (Hudson, McDonough et al., 2018; McDonough et al., 2019). Here, no such prior information was available. The actions started from a neutral position and the goals only became apparent once the action was underway, from the subtle preshaping of the hands to the affordances of the goal object (i.e., precision grip when directed toward the small object, power grip when directed toward the large object).

Our results therefore show, first, that predictions are not just made before action onset, but are dynamically adjusted “online” as more information becomes available from the unfolding kinematics and are then integrated with the action’s perceptual representation. Second, they reveal that matching of actions to the affordances of potential goal objects in the environment plays a major role in this process, as previously hypothesized (see Bach et al., 2014 for a theoretical proposal and review). Third, they go beyond prior work that has shown that people use such affordance matching to identify the target of another’s action, guiding eye movements toward it in an anticipatory manner (e.g., Ambrosini et al., 2011, 2013; Bach et al., 2011). They reveal that these predictions do not just represent the likely goal object, but represent concrete expectations about the next step of a hand’s path through the scene, which are perceptually integrated with the actually observed kinematics.

A surprising finding for such theories of top-down guided predictive perception was that this matching of actions to goal objects and the resulting perceptual biases appeared to be highly automatic and independent from explicit judgments about the action’s goals. We had hypothesized that if perceptual mislocations and explicit judgments inform each other, then those trials that were explicitly judged to be directed toward the top object should also show upward mislocations, and vice versa for judgments toward bottom objects. However, converging findings from three independent tests argue against this interpretation. First, in Experiment 1, the action’s goal was highly task relevant because participants indicated verbally, after each action, which object they believed was the target. No such response was required in Experiment 2, such that any perceptual bias indexes only spontaneous, implicit goal inferences and predictions that are nevertheless made by participants. Nevertheless, the perceptual bias toward the grip-matching object was evident—with virtually identical effect sizes—in both experiments. Second, in Experiment 1, correlational analyses across participants showed that the perceptual biases were independent of whether participants’ verbal goal judgments revealed a reliance on grip information or not. Thus, the perceptual bias toward the matching goal object was of similar size irrespective of whether participants made use of grip–object matching in their explicit postaction judgments. Third and finally, correlational analyses across trials that directly relate perceptual displacements in a given trial to verbal goal judgments in the same trial (Experiment 1) confirmed this lack of a top-down influence. Even though the actual goal object predicted both the direction of the perceptual bias and which object was explicitly reported as a target, the perceptual biases and verbal goal judgments remained uncorrelated across trials.

Together therefore, the results of these three independent tests show that, while the mechanisms for action prediction and the goal identification both rely on grip–object matching, the two mechanisms do not strongly inform each other: explicit goal judgments do not induce perceptual biases, nor do perceptual biases induce explicit goal judgments. While it had been difficult to draw conclusions from null effects, recent Bayesian and equivalence-testing (TOST) analysis techniques that were used here have overcome this problem (Lakens, 2017). Indeed, all three tests described above provide substantial to strong evidence for the *absence* of a link between explicit judgments and perceptual biases, and the reported difference remain significantly within equivalence bounds.

This apparent dissociation between explicit and implicit perceptual biases is in line with prior reports of similar dissociations during motion perception in representational momentum task, where people’s abstract knowledge of an object’s future course deviates from their perceptual predictions (e.g., Freyd & Jones, 1994; Kozhevnikov & Hegarty, 2001). It may appear surprising, however, from a viewpoint of recent predictive coding accounts, according to which predictions and prediction errors ensure that top-level and lower-level judgments remain aligned (Clark, 2013; Friston & Kiebel, 2009). Thus, any inferences on a higher level—for example, what the goal of the action is—would propagate downward to lower levels and inform perceptual judgments. Conversely, any change in perceptual estimation—whether the hand is perceived to travel upward and downward—would, via prediction errors, inform resulting high-level judgments of action goals.

This apparent conflict can be resolved if one accepts recent proposals that predictions can also emerge locally, from top-down interactions within the human perceptual system for the perception of biological motion (for a review, see Scholl & Gao, 2013), without drawing on information external to these networks such as high-level explicit action goal judgments (e.g., Firestone & Scholl, 2016). Indeed, several lines of evidence suggest that the perceptual system itself can detect many aspects of intentional behavior, without the need for higher-level evaluation, such as whether one actor chases another (Gao, McCarthy, & Scholl, 2010) or whether actors pay attention to their reach or the target (e.g., Jellema, Baker, Wicker, & Perrett, 2000). The match between hand and goal object may therefore provide another feature from which such lower-level teleological interpretations of observed motion can be derived, and low-level perceptual regions have indeed been found to be sensitive to such information (e.g., Superior temporal sulcus, Gao, Scholl, & McCarthy, 2012; Saxe, Xiao, Kovacs, Perrett, & Kanwisher, 2004; lateral occipital cortex, Bracci & Peelen, 2013). Our new data then suggests that these regions are not only sensitive to the presence of these matches but that they also use them to predict the action’s further path and bias the perceptual representations toward it, independently of the goals explicitly attributed to the other person.

Future studies now need to investigate from what kind of mechanism the perceptual biases emerge. Our prior studies point toward lower-level perceptual processes that determine participants’ conscious perceptual experience of the actions, which then drives their explicit judgments. Several studies from our lab (Hudson, Bach et al., 2018; Hudson, Nicholson, Ellis et al., 2016; Hudson, Nicholson, Simpson et al., 2016) have shown, for example, that the type of perceptual displacements measured here emerge not only with touchscreen judgments, which may also have a motor (pointing) component, but also purely perceptual judgments, in which participants compare the hand disappearance points with probes in the same, future, or prior position. Even when these probes were presented only 250 ms after hand disappearance, the same perceptual distortions were apparent, suggesting at the very least an effect in iconic memory. Second, in our most recent work testing perceived changes to action kinematics in the presence of obstacles, all such effects were eliminated when dynamic visual noise masks were presented briefly (560 ms) between action offset and touchscreen response (Hudson, McDonough et al., 2018), which are known to interfere with reentrant top-down projections to early visual cortex (Fahrenfort, Scholte, & Lamme, 2007; Lamme, Zipser, & Spekreijse, 2002).

Together, therefore, these findings support a low-level locus of the effects that either reflects the top-down sharpening of perceptual representations during motion perception (i.e., motion blurring, Hammett, 1997), or the filling-in of the expected path after the unexpected sudden offset (Ekman et al., 2017). Neuroimaging studies would be useful to disentangle to what extent the perceptual changes we have measured here reflect changes in early perceptual systems, similar to that seen in various visual illusions and sometimes in motion illusions (e.g., apparent motions, Muckli, Kohler, Kriegeskorte, & Singer, 2005; predicted motion preplay, Ekman et al., 2017).

Another challenge is elucidating whether—and via which mechanisms—the perceptual predictions ultimately influence cognition and behavior. On first glance, the relatively small absolute size of the effects (∼1 pixel) may suggest an only negligible influence in real-life social interactions. However, this needs to be evaluated against the background of a not very salient stimulus feature that is available only late during action observation, and that, even when participants’ were explicitly asked, only induced a 17% shift away from chance performance. On this view, it is striking that robust shifts in perceptual experience are observed at all, with an effect size that is comparable to other effects in psychology (Schäfer & Schwarz, 2019). Indeed, in our prior work in which the decks were not stacked against finding perceptual biases and the prediction-relevant stimulus features were available earlier and much more salient (e.g., the spatial setup of the scenes, intention statements of the actors), the effects were much larger, between *d* = .52 and *d* = 1.23 (e.g., Hudson, Nicholson, Ellis et al., 2016; Hudson, Bach et al., 2018; McDonough et al., 2019).

The question about how the measured biases would manifest in real-life social interactions is also complicated by the fact that there is still considerable debate about how, specifically, predictions would influence behavior, with different theoretical frameworks making different predictions. For example, in Bayesian-perception views, predictions primarily serve to “sharpen” ambiguous social perception (e.g., Yon, Gilbert, de Lange, & Press, 2018; Kok, Jehee, & de Lange, 2012) or to “fill in” missing or ambiguous stimulus aspects (e.g., during occlusion or when saccading away). The influence of such a sharpening depends inversely on the reliability one attributes to the real input. In contrast to here, in real-life situations, participants do not closely watch the actions and report their spatial extent. They see actions often only briefly before saccading away (e.g., toward the anticipated goal object), they plan their own responses at the same time, and the actions take place in crowded environments with unpredictable onsets. All these factors would conspire to reduce attention to the seen movements, thereby decreasing their reliability and in turn increasing the reliance of top-down predictions beyond what is measured in our experiments.

In other views, predictions do not primarily serve perceptual functions, but to help “bridge the gap” between relatively slow perceptual and quick, online motor control processes (Hubbard, 2006, see also Müsseler, Stork, & Kerzel, 2008; Nijhawan, 1994, 2008). In such a view, the weight given to expectations increases the more the observed action needs to provide a control signal for action control (e.g., intercepting an observed reach), and their effect is best measured not in resulting perceptual distortions but in anticipatory changes to these actions. Indeed, several studies have shown that perceptual distortions can have different—and usually larger—effects when measured directly on the actions directed at these stimuli (e.g., Brouwer & Knill, 2007, 2009), and that perceptual biases themselves increase the more they serve as control signal for the observer’s actions (Kerzel, 2003; Müsseler et al., 2008).

Finally, recent predictive-processing approaches assume that the primary role of predictions is neither to primarily serve perception nor to guide action (even though both are relevant contributions), but to perceptually test one’s prior assumptions about the other person against their actual behavior, so that in the long run one’s knowledge of the (social) world remains aligned with reality (e.g., Clark, 2013; Friston & Kiebel, 2009; see Bach & Schenke, 2017, for an application to social perception). In such views, the subtle biases in visual judgments measured in our paradigm show that such perceptual comparisons are being made, but they say nothing about their ultimate role in shaping social judgments, when an action clearly mismatches the prior predictions.

Together, therefore, the perceptual distortions measured here and in our prior work show that people make predictions about the course of others’ actions, and that these predictions are available in a format that is dynamically integrated with the perceptual input, even when the action is already underway. They may therefore provide a promising starting point in disentangling these not mutually exclusive roles that perceptual predictions could play in human interactions with the (social) world.

## Conclusions

The present results reveal that the perceptual experience of others’ actions is predictively shaped by the integration of the unfolding action kinematics with the affordances of available goal objects, as proposed by recent predictive accounts of social perception (Csibra, 2008; Kilner et al., 2007a, 2007b; Bach et al., 2014; Bach & Schenke, 2017). These integrations likely emerge at a relatively low level, from processes within systems for the perception of biological motion, without influences from top-down evaluations of others’ goals and intentions. Future studies must now resolve precisely via which mechanism predictions act on perceptual representations, how they help guide one’s own actions toward future states in social interactions, and how prior knowledge is updated and revised if it consistently fails to explain the perceptual input.

## Supplementary Material

10.1037/xhp0000745.supp

## Footnotes

1 Please note that although our study design is based on prior work on Representational Momentum (e.g. Hubbard, 2005; Hubbard & Bharucha, 1988), the predicted effects are not along the trajectory of motion (the X-axis), typically referred to as “M-Displacement.” Instead, perceptual biases are predicted along the axis perpendicular to the direction of motion and towards the apparent target object (the Y-axis). These displacements are typically referred to as “O-Displacement.” We first calculate perceptual biases as differences in screen coordinates between the actual stimulus location and participant’s touchscreen responses, making both the apparent shifts towards the relevant goal object (along the Y-axis) and the general shift towards the hands’ center of gravity (along the X-axis) directly visible. We then provide a summary measure of the predicted perceptual displacements on the Y-axis (“O-Displacement”), reported in Figure 2C.
