# Supplementary material for: Affordance Matching Predictively Shapes the Perceptual Representation of Others’ Ongoing Actions
Source: J Exp Psychol Hum Percept Perform. 2020 May 7;46(8):847–59. doi: 10.1037/xhp0000745 (PMC7391862; doi:10.1037/xhp0000745)
Supplement: Supplementary file 1 [file Supplementary_Table_xhp0000745.docx]

Supplementary Table 1.

|  |  |  |  |  |  |  |  |  |  |
| --- | --- | --- | --- | --- | --- | --- | --- | --- | --- |
|  | X-axis | | | |  | Y-axis | | | |
| Measures | *F* | *p* | *η_p_^2^* | BF10 |  | *F* | *p* | *η_p_^2^* | BF10 |
| Object Location | .289 | .592 | .002 | .103 |  | .763 | .384 | .006 | .103 |
| Object Location * Experiment | 4.07 | .046 | .032 | .185 |  | 1.62 | .205 | .013 | .152 |
| Grip Type | 503 | .000 | .804 | 1.17E+99 |  | 1130 | .000 | .902 | 3.20E+141 |
| Grip Type * Experiment | .113 | .738 | .001 | .171 |  | 1.04 | .310 | .008 | .472 |
| Object Location * Grip Type | .012 | .913 | .000 | .138 |  | 16.8 | .000 | .120 | 158 |
| Object Location * Grip Type * Experiment | 1.17 | .281 | .009 | .226 |  | .666 | .416 | .005 | .263 |

*All effects from the 2x2x2 mixed ANOVA, with Grip type (power vs precision) and Object location (large target on top vs small target on top) as repeated measures factors and Experiment (1: explicit prediction vs 2: implicit prediction) as between-subjects factor. The table also includes Bayes Factors.*
